# Supplementary material for: The causal role of circulating inflammatory markers in osteoporosis: a bidirectional Mendelian randomized study
Source: Front Immunol. 2024 Jul 18;15:1412298. doi: 10.3389/fimmu.2024.1412298 (PMC11291241; doi:10.3389/fimmu.2024.1412298)
Supplement: Supplementary file 1 [file DataSheet_1.zip › supplementary material/OSTEOPOROSIS-CIM/GCST90274786.tsv.gz_leave_one_out.pdf]

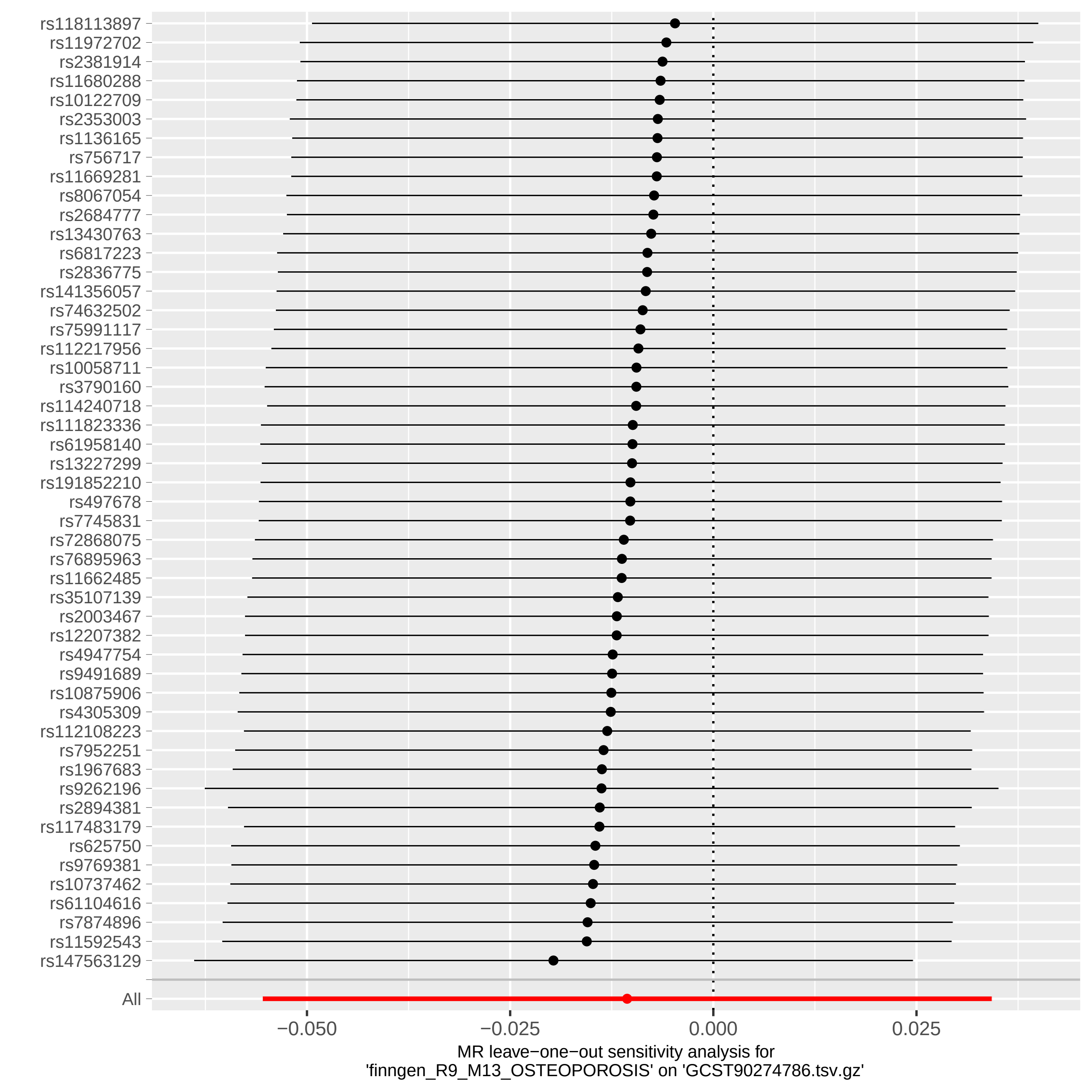
rs118113897
rs11972702
rs2381914
rs11680288
rs10122709
rs2353003
rs1136165
rs756717
rs11669281
rs8067054
rs2684777
rs13430763
rs6817223
rs2836775
rs141356057
rs74632502
rs75991117
rs112217956
rs10058711
rs3790160
rs114240718
rs111823336
rs61958140
rs13227299
rs191852210
rs497678
rs7745831
rs72868075
rs76895963
rs11662485
rs35107139
rs2003467
rs12207382
rs4947754
rs9491689
rs10875906
rs4305309
rs112108223
rs7952251
rs1967683
rs9262196
rs2894381
rs117483179
rs625750
rs9769381
rs10737462
rs61104616
rs7874896
rs11592543
rs147563129
All
−0.050
−0.025
0.000
0.025
MR leave−one−out sensitivity analysis for
'finngen_R9_M13_OSTEOPOROSIS' on 'GCST90274786.tsv.gz'
